# Morphological and bioenergetic demands underlying the mitophagy in post-mitotic neurons: the pink–parkin pathway

**DOI:** 10.3389/fnagi.2014.00018

**Published:** 2014-02-18

**Authors:** Giuseppina Amadoro, Veronica Corsetti, Fulvio Florenzano, Anna Atlante, Antonella Bobba, Vanessa Nicolin, Stefania L. Nori, Pietro Calissano

**Affiliations:** ^1^Institute of Translational Pharmacology – National Research CouncilRome, Italy; ^2^European Brain Research InstituteRome, Italy; ^3^Institute of Biomembrane and Bioenergetics – National Research CouncilBari, Italy; ^4^Clinical Department of Medical, Surgical and Health Science, University of TriesteTrieste, Italy; ^5^Department of Medicine and Surgery, University of SalernoBaronissi, Italy

**Keywords:** mitochondria dynamics, quality control, mitophagy, primary neurons, neurodegenerative diseases

## Abstract

Evidence suggests a striking causal relationship between changes in quality control of neuronal mitochondria and numerous devastating human neurodegenerative diseases, including Parkinson’s disease, Alzheimer’s disease, Huntington’s disease, and amyotrophic lateral sclerosis. Contrary to replicating mammalian cells with a metabolism essentially glycolytic, post-mitotic neurons are distinctive owing to (i) their exclusive energetic dependence from mitochondrial metabolism and (ii) their polarized shape, which entails compartmentalized and distinct energetic needs. Here, we review the recent findings on mitochondrial dynamics and mitophagy in differentiated neurons focusing on how the exceptional characteristics of neuronal populations in their morphology and bioenergetics needs make them quite different to other cells in controlling the intracellular turnover of these organelles.

## INTRODUCTION

Mitochondria exhibit dynamic properties (fusion, fission, transport, biogenesis, and degradation) and their homeostasis in a healthy, functional network is a process which involves an intimate crosstalk between quality control and selective autophagy (Twig et al., 2008; Westermann, 2012). All these events are closely coordinated and reciprocally interact in an integrated system, constantly monitored in every cell to maintain a well-performing mitochondrial population which can sustain a proper bioenergetic status and, consequently, viability. The quality control of mitochondria relies on several different pathways, including: (i) degradation of misfolded proteins located in the matrix and intermembrane space by intrinsic mitochondrial proteases; (ii) clearance by ubiquitin–proteasome systems (UPS) of damaged proteins located in outer membrane or of nuclear-coded mitochondrial proteins which are not correctly imported; (iii) removal of oxidated proteins/lipids by mitochondria-derived vesicles which are directly targeted for lysosomal or peroxisomal degradation. Besides, recent studies have uncovered a novel autophagic process addressed selectively toward mitochondria and named mitophagy, which is – up to date – the only identified mechanism by which these defective and malfunctioning organelles are entirely recycled or degraded. Remarkably, mitophagy can also play a key physiological role by providing for the developmental maturation of reticulocytes as well for the exclusive maternal inheritance of mt DNA upon oocytes fertilization (Ding et al., 2012; Ashrafi and Schwarz, 2013).

The classical regulation of autophagy is governed by the mammalian target of rapamycin (mTOR) pathway, which negatively controls this process. The serine/threonine kinase mTOR belongs to the phosphatidylinositol kinase-related kinase (PIKK) family and is in charge of important intracellular processes such as translation, metabolism, and transcription in response to nutrients and/or growth factors (Sarkar, 2013). In mammalian cells, mTOR binds several proteins to form two distinct protein multi-complexes. mTORC1 (mTOR complex 1) contains the scaffolding protein Raptor and additional proteins that also function in association with TOR in a second complex, called mTORC2 (mTOR complex 2), that is not directly involved in autophagy. mTORC1 is a key regulator of translation and ribosome biogenesis as well is responsible for autophagy induction in response to starvation (Ghavami et al., 2014). On the other hand, although being originally reported as rapamycin-insensitive, mTORC2 is also likely targeted by this drug and is involved in the regulation of phosphorylation and activation of Akt/PKB, protein kinase C, serum- and glucocorticoid-induced protein kinase 1. In addition, as Akt positively regulates mTORC1, it would be possible that mTORC2 also acts as a negative regulator of autophagy (Jung et al., 2010) Accumulating data also highlight the crucial role of mTOR-dependent signaling pathways in neurodegenerative diseases, including Huntington’s disease (HD), Parkinson’s disease (PD), and Alzheimer’s disease (AD; Sarkar, 2013), as its pharmacological inhibition by rapamycin attenuates the accumulation of misfolded/aggregated proteins and protects against neuronal loss, *in vivo* and *in vitro* (Ghavami et al., 2014). Nevertheless, as compelling evidence demonstrate that canonical non-selective autophagy and mitophagy share the similar core autophagosome/mitophagosome initiation and formation machinery (Hayashi-Nishino et al., 2009; Yoshii et al., 2011), a two-step mitophagy model has been proposed to occur in mammalian cells involving the initial induction of autophagy-related genes (Atg)-dependent macroautophagy followed by the mitochondrial priming. In the first step, the reactive oxygen species (ROS) accumulation and the ATP depletion (indirectly via AMPK, AMP-activated protein kinase, activation) as result of damaged and dysfunctional mitochondria, inhibit the induction of mTOR which under physiological conditions blocks the autophagy by restraining the kinase activity of ubiquitin-like kinase (ULK; Laplante and Sabatini, 2009; Ghavami et al., 2014). Upon escaping from mTOR suppression, the ULK complex, including ULK-1 (mammalian Atg1 ortholog), Atg13, Atg101, and FIP200 promotes the *de novo* formation of the initiation complex by regulating the activity of the class III phosphoinositide-3 kinase (PI3K) including Beclin-1 (mammalian Atg6), Atg14, Ambra1 (activating molecule in Beclin-1-regulated autophagy), vacuolar protein sorting 34 (Vps34), and Vps15, to form PI3P, which further recruits several PI3P-binding proteins to drive the formation of the initiation membrane. The Atg12–Atg5–Atg16L1 multi complex and LC3 (microtubule-associated protein 1A/1B-light chain 3)–PE (phosphatidylethanolamine) conjugates are later involved in the elongation and closure of the initiation membrane (Itakura and Mizushima, 2010; Feng et al., 2013; Sarkar, 2013). In the second step, the priming of mitochondria is mediated by different mechanisms that could be Parkin-dependent, involving the Parkin–Pink1-mediated pathway (Youle and Narendra, 2011), or Parkin-independent, involving the ubiquitin E3 ligase SMURF1 (Orvedahl et al., 2011), the outer mitochondrial membrane (OMM) protein Nix (Schweers et al., 2007; Sandoval et al., 2008; Novak et al., 2010) and FUNDC1 (Liu et al., 2012a), the HSP90–Cdc37 chaperone complex stabilizing and activating ULK-1 (Joo et al., 2011) and the Atg9A/ULK-1 complex (Itakura et al., 2012). An Atg-independent mitophagy, involving the 15-lipoxygenase, has been also described (van Leyen et al., 1998) but the precise role of this enzyme in organelles degradation is still not completely clarified.

**FIGURE 1:**
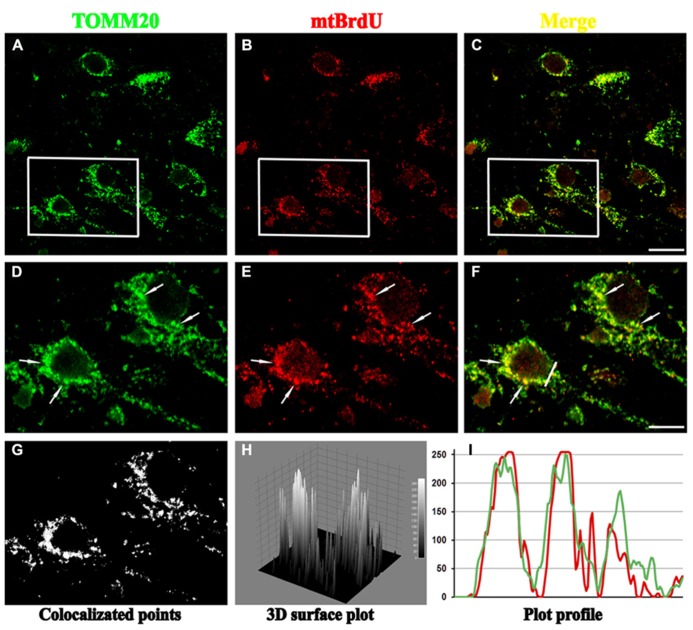
**Confocal microscopy of mature hippocampal neuronal culture (15 DIV) double stained for TOMM20 (a mitochondrial marker; green channel) and BrdU (for visualization of newly synthesized mito-chondrial DNA; red channel).** BrdU puncta colocalize with several TOMM20 positive mitochondria (arrows) in cell body, showing that biogenesis of these organelles mainly occurs in perinuclear compartment. **(A–C)** Lower magnification of TOMM20/BrdU immunofluorescence. **(D–F)** Inset higher magnification. **(G–I)** Colocalization analysis performed with ImageJ, including the spatial pattern of colocalized points **(G)**, the luminance intensity height of the colocalized points **(H)**, the spatial intensity profile for both fluorescence channels **(I)** of the white line positioned in **(F)**. Scale bar 15 and 5 μm. Figure referring to data from Amadoro et al. (2014).

Moreover, as the mitophagy pathway has been mainly studied in non-neuronal cell lines, this process is still not fully clarified in terminally differentiated neurons. In polarized neurons mitochondria have a longer half-life than in other post-mitotic tissues (Menzies and Gold, 1971; Miwa et al., 2008; O’Toole et al., 2008) and, although the translation of a subset of mitochondrial proteins may occur in axons (Kaplan et al., 2009), the import of most of those newly synthesized that are stably localized on these organelles occurs in the cell body followed by their transport toward distal axons, dendrites and synaptic sites. Removal of damaged mitochondria is as well a bioenergetically demanding task for neuronal populations because these organelles need to be actively retro-transported to the cell body in order to fuse with locally resident lysosomes (Wang et al., 2006). Besides, although physiological aging has been associated with decreased mitochondrial functions and with mitophagic processes (Batlevi and La Spada, 2011; Green et al., 2011), functional as well as morphological impairment of these organelles – especially for those neuronal populations with poorly myelinated, long, thin axons located in selective brain areas (Verstreken et al., 2005) – have been causally connected to several human neurological disorders (Lin and Beal, 2006; Johri and Beal, 2012). An unbalanced turnover, recycling/elimination of the entire mitochondria through selective autophagy is indeed considered an early event involved in the pathogenesis of Charcot–Marie–Tooth (CMT) disease, PD, AD, HD (Lin and Beal, 2006; Wang et al., 2009; Batlevi and La Spada, 2011; Imai and Lu, 2011; Karbowski and Neutzner, 2012; Nunnari and Suomalainen, 2012; Sheng and Cai, 2012; Chaturvedi and Beal, 2013; Itoh et al., 2013), amyotrophic lateral sclerosis (ALS; Cozzolino and Carrì, 2012), cerebral ischemic models (Calo et al., 2013), schizophrenia, and depression (Deheshi et al., 2013). To this regard, it is worth mentioning that patogenetic and/or misfolded/aggregated proteins such as mutated superoxide dismutase in ALS (Israelson et al., 2010), mutant huntingtin in HD (Rockabrand et al., 2007), β-amyloid (Aβ), and tau in AD (Caspersen et al., 2005; Manczak et al., 2006; Hansson Petersen et al., 2008; Amadoro et al., 2010, 2012; Du et al., 2012; Schmitt et al., 2012), mutant α-synuclein in PD (Devi et al., 2008) *in vivo* accumulate on mitochondria causing their functional decline which, in turn, may affect their mitophagic elimination (Moreira et al., 2007; Chinta et al., 2010; Choubey et al., 2011; Amadoro et al., 2014; **Figure 1**). Furthermore, any direct change in the mitochondrial biogenesis pathway [peroxisome proliferator-activated receptor α coactivator/nuclear respiratory factor 1 (PGC-1α/NFR-1)] in neurons may also contribute itself to accumulation of the above-mentioned pathogenetic proteins at these organelles – affecting consequently their metabolic functions – as recently shown for the long insulin-degrading enzyme (IDE) isoform which is involved in mitochondrial Aβ catabolism (Leal et al., 2013).

Interestingly, the relevance in assuring a proper quality control of neuronal mitochondria clearly comes into view considering that the mitochondrial dynamics which occur in a restricted hypothalamic populations can exert systemic effects and contribute to the physiological response of the whole-body energetic balance. To this regard, two recent papers provide striking evidence that alterations in mitochondrial number and morphology (Dietrich et al., 2013) or in mitochondria–endoplasmic reticulum (ER) connectivity (Schneeberger et al., 2013), both respectively occurring in orexigenic [agouti-related peptide (Agrp)- and neuropeptide Y (NPY)-producing] and antiorexigenic [pro-opiomelanocortin (POMC)-producing] hypothalamic neurons, allow the metabolic adaptation of feeding mice to different diet conditions.

In the present review, we focus on several specific and peculiar aspects of the mitochondrial clearance mediated by the Pink1–Parkin pathway in neurons and on the pathological implications that their inappropriate regulation may have in the onset and/or progression of human neurodegeneration. Furthermore, we will take advantage of the fact that a growing body of information on the mechanisms involved in mitophagy in non-neuronal cells has been extensively reported by other authors (Detmer and Chan, 2007; Palmer et al., 2011; Wang and Klionsky, 2011; Ashrafi and Schwarz, 2013).

## REGULATION OF MITOCHONDRIAL DYNAMICS AND BIOENERGETIC STATUS IS OF PARTICULAR RELEVANCE TO POST-MITOTIC NEURONS

**FIGURE 2:**
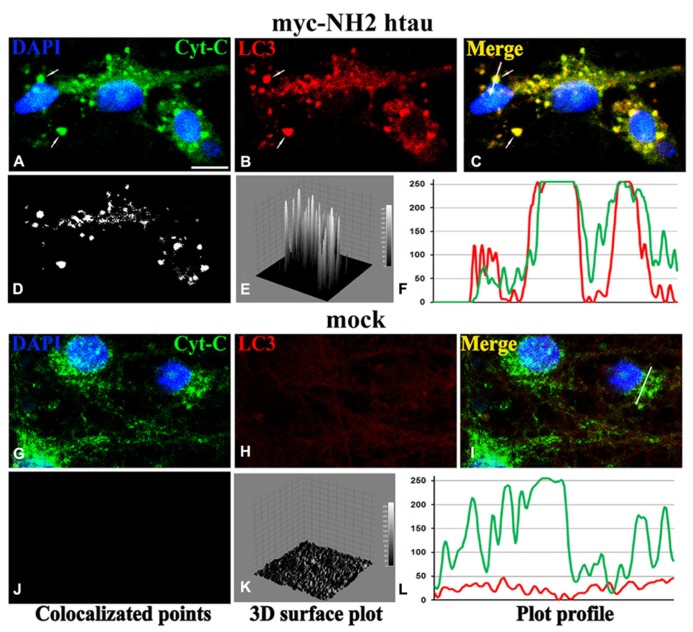
**Confocal microscopy and image analysis of double immunofluorescence for cyt C (a mitochondrial marker; green channel) and LC3 (for visualization of autophagosomes; red channel), carried out on primary mature hippocampal cultures (15 DIV) at 12 h post-infection (MOI 50) with mock- and myc-NH_2_ 26-230 human tau vectors.** Nuclei were stained with DAPI (blue channel). **(A–C)** Numerous labeled LC3 stained vesicles intensely positive (colocalized) for cyt C were observed in myc-NH2htau neurons. Arrows point to two large mitophagosomal structures. **(G–I)** LC3 immunofluorescence is very faint in mock-treated cultures **(D–F)**; **(J–L)** colocalization analysis performed with ImageJ, including the spatial pattern of colocalized points of **(D,J)**, the intensity height of the luminance in the colocalized points **(E,K)**, the spatial profile of the fluorescence intensity for both fluorescence channels for the white line positioned on several mitophagosomes in the second row **(F,L)**. Scale bar 7 μm. Figure referring to data from Amadoro et al. (2014).

The mitochondria quality control regulates in post-mitotic neurons several vital metabolic functions such as their proper distribution to synaptic terminals, maintenance of electron transport chain (ETC) activity and electrical connectivity (Stowers et al., 2002; Verstreken et al., 2005; Chen and Chan, 2006; Liu and Shio, 2008; Bereiter-Hahn and Jendrach, 2010; Ferree and Shirihai, 2012; Misko et al., 2012), protection of mtDNA integrity (Westermann, 2002; Parone et al., 2008), apoptosis (Suen et al., 2008), formation and function of synapses and dendritic spines (Li et al., 2004). As other cell types, neuronal populations also continually modulate size and number of these organelles, according to the variable energy demands and metabolic states throughout the entire lifetime and/or different sub-cellular compartments (Chen and Chan, 2009; Santos et al., 2010; Vives-Bauza and Przedborski, 2011; Van Laar and Berman, 2013). However, a tight control of the interplay between their mitochondrial dynamic and bioenergetic status is of particular relevance for post-mitotic neurons because they have a unique metabolic as well as morphological profile which entails specialized and compartimentalized energetic needs. Indeed, neuronal populations are typically characterized by: (i) high bioenergetic needs as the ATP production classically depends in these cells on OxPhos respiration rather than glycolysis (Rolfe and Brown, 1997; Attwell and Laughlin, 2001; Mironov, 2009; Bolanos et al., 2010); (ii) a very polarized morphology with extensive neuritic projections which are crucial for neuronal survival via a proper maintenance of their mitochondrial biomass. Remarkably, although the human brain consists of only 2% of the volume of the body, it is roughly responsible for 25% of net oxygen consumption in resting conditions (Magistretti and Pellerin, 1999) with neurons generating as much as 95% of their ATP exclusively from mitochondrial OXPhos (Erecińska et al., 1994). However, it is worth noticing that neurons and astrocytes – which are the two major types of brain cells – exhibit a different preference for glucose utilization since its metabolism in neuron is diverted mainly to the pentose phosphate pathway in order to regenerate antioxidants (reduced glutathione) and to promote survival (Bolanos et al., 2010). As they constantly require an active and efficient defense mechanism against oxidative stress (Almeida et al., 2005; Herrero-Mendez et al., 2009), neuronal populations are indeed unable to switch to anaerobic glycolytic metabolism (as an ATP-generating mechanism) during an acute mitochondrial stress, relying on lactate as an alternative substrate for their mitochondria-derived bioenergetic purposes (Pellerin et al., 2007). In addition, it is generally assumed that only 0.2% of the neuronal cellular volume is in the cytoplasmic soma while about 99.8% is constituted by the axonal and dendritic compartments (Devor, 1999; Fjell and Walhovd, 2010; Florenzano, 2012). For instance, the axonal length of projection neurons – such as those of rat dopaminergic neurons that are localized in the *substantia nigra* which is an area selectively affected in PD – can be as long as 470 μm or more and can provide several axon collaterals each of them making in turn contacts with approximately 400 synapses (Matsuda et al., 2009). Besides, in order to locally provide ATP supply and calcium buffering required for the neuronal activity of high energy-demanding terminal synapses (Schon and Przedborski, 2011), an high number of mitochondria resides far away from soma being localized in distal axon and dendritic processes (Hollenbeck, 2005) such as presynaptic terminals, including the active zones where synaptics vesicles (SVs) are released (Rowland et al., 2000; Perkins et al., 2010), post-synaptic densities, nodes of Ranvier and in growth cones (Fabricius et al., 1993; Morris and Hollenbeck, 1993). Collectively, such peculiar requirements imply that a high number of mitochondria spend the majority of their time in traveling up-and-down between the sites of their biogenesis, which are mainly localized into cell bodies (Davis and Clayton, 1996; Saxton and Hollenbeck, 2012; **Figure 2**), and those of their functional utilization which instead are close to terminal endings (Li et al., 2004). Interestingly there’s a positive correlation between the frequency and/or the intensity of synapse electrical activity and the number of metabolically active mitochondria at presynaptic compartment (Dubinsky, 2009). Furthermore, provided that the mitochondria size and mass are not the same for all neurons (Dubinsky, 2009; Lu, 2009) and that intrinsic mitophagic capacity has been found to be brain region-specific (Diedrich et al., 2011), any perturbation in controlling the dynamics properties of these organelles (Verstreken et al., 2005; Kann and Kovács, 2007; Nunnari and Suomalainen, 2012) can seriously and selectively compromise their survival (Xue et al., 2001). Different morphologies and ultrastructural profiles of mitochondria have been also correlated with distinct bioenergetic demands of the tissues they occupy and mitochondrial network in neurons is demonstrated to be distinct from those of other tissues in morphology, interconnectivity as well as cytoplasmatic pattern distribution (Dubinsky, 2009; Kuznetsov et al., 2009; Mironov, 2009). Neurons critically depend on autophagy for differentiation and survival (Komatsu et al., 2006) and are particularly prone to autophagic stress (Chu, 2006) so that basal autophagy appears to be more efficient than in other proliferating non-neuronal cells types (Boland et al., 2008) and is also likely to be regulated in alternative and quite different ways (Ashrafi and Schwarz, 2013; Van Laar and Berman, 2013). Being terminally differentiated and producing high levels of ROS against relatively fewer antioxidant molecules (Cui et al., 2004; Fatokun et al., 2008), neurons need to refurbish “old” mitochondrial pool to prevent the deleterious accumulation of oxidative damage, as suggested by the fact that antioxidant treatment supports the survival of cerebellar Purkinje cells (PCs) from knockout mice for dynamin-1-like protein (Drp1) fission protein (Kageyama et al., 2012). In contrast, proliferating cells constantly generate “new” mitochondria during continuous cell replication cycles so that oxidative stress may be diluted and maintained at relatively low levels, even without any mitochondrial division (Kageyama et al., 2012). A proper interplay between the bioenergetic – which is mainly regulated by energy requirement and substrate availability – and the mitochondrial quality control and autophagy – which control the overall health of mitochondrial population and their relative abundance – is specifically critical in neurons as it impinges on their intrinsic resistance to stress and, then, on their survival. Indeed, the relative sensitivity of different neuronal populations to mitochondrial inhibitors is tightly correlated with their “spare respiratory capacity,” which is an index of general mitochondrial health taking into account the ratio of the glucose utilization rate to the expression level of respiratory chain complexes (Fern, 2003). Interestingly, the vulnerability to a complex I inhibitor such as rotenone of cultured striatal neurons, which are selectively affected in HD pathology, is primarily determined by their “spare respiratory capacity” rather than oxidative stress (Yadava and Nicholls, 2007). Likewise, presynaptic mitochondria from hippocampus and cortex show a lower spare respiratory capacity to rotenone when compared to non-synaptic mitochondria from the same regions (Davey et al., 1997), in favor with the finding that the bioenergetic failure of peripheral mitochondria *in vivo* initiates the loss of synaptic terminals in neurodegenerative diseases.

Nevertheless, despite the regulation of the physiological mitochondrial turnover is of particular importance for neuronal populations, it is not completely clear, as yet, whether an up-regulation or a down-regulation of mitophagic processes accompanied to an uncoordinated biogenesis can critically contribute to the onset and/or progression of human neurodegeneration (Batlevi and La Spada, 2011; Zhu et al., 2012, 2013).

## THE PINK/PARKIN PATHWAY IN NEURONS: ENERGETIC METABOLISM, NATURE, AND SEVERITY OF INJURY DICTATE THE REGULATION OF MITOCHONDRIAL DEGRADATION IN MATURE NEURONS

**FIGURE 3:**
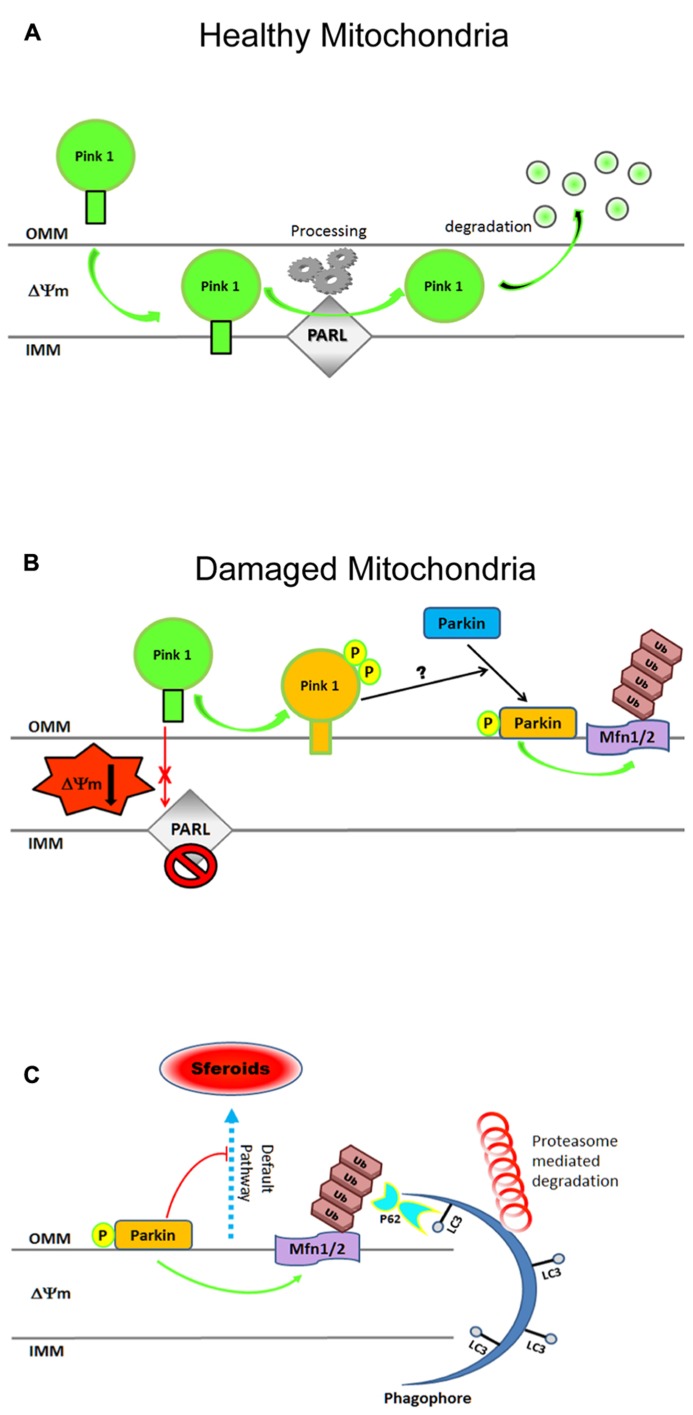
**Cartoon illustrating steps in the mitochondrial clearance mediated by the Pink1–Parkin pathway. (A)** In physiological conditions, Pink-1 is constitutively imported into healthy mitochondria via TIM/TOM complex to the inner membrane (IMM), cleaved by presenilin-associated rhomboid-like protease (PARL), and then proteolytically degraded. **(B)** Upon ΔΨ collapse, full-length Pink1 is not processed accumulating at the outer membrane (OMM) to recruit Parkin onto depolarized mitochondria. PINK1 autophosphorylation at Ser228 and Ser402 (P) is essential for efficient mitochondrial localization of Parkin. The PINK1-dependent Parkin phos-phorylation at Ser65, combined with unknown factor(s) (?), is required not only for its efficient translocation but also for the degradation of mito-chondrial proteins during mitophagy. **(C)** After being recruited on OMM, Parkin triggers mitophagy by ubiquitylating (Ub, K48, K63) several proteins including Mfns1/2, VDAC, TOM. Proteasome-mediated removal of Mfns1/2 not only inhibits mitochondrial fusion but also prevents its *default* rear-raggedright rangement into spheroids, allowing thus the damaged organelles to be recognized by the engulfing autophagosome. Cytosolic autophagy adaptor p62 (also known as sequestosome 1, SQSTM1) is also involved in mito-phagy as its K63-ubiquitin-binding domain (UBA) as well as an LC3- binding domain (LIR), recruits autophagosomes to ubiquitylated protein. For more information on Pink–Parkin-dependent mitophagy, please refer to recent excellent reviews (Twig and Shirihai, 2011; Vives-Bauza and Przedborski, 2011; Youle and Narendra, 2011; Ding and Yin, 2012; Jin and Youle, 2012). Freely adapted from Figure 6 of (Okatsu et al., 2012).

The Pink/Parkin pathway involves the interplay of two recessive Parkinson’s-linked genes PTEN-induced kinase 1 (PINK1) – a mitochondrially targeted serine/threonine kinase – and Parkin – an E3 ubiquitin ligase – which cooperate in maintaining the mitochondrial integrity by regulating several physiological processes of these organelles, including their membrane potential, calcium homeostasis, cristae structure, respiratory activity, and mtDNA integrity (Trempe and Fon, 2013). In addition, the Pink/Parkin pathway is crucial for autophagy-dependent clearance of dysfunctional mitochondria (Narendra et al., 2008; Matsuda et al., 2010; **Figure 3**) as, in the absence of PINK1 or Parkin, cells often develop fragmented mitochondria (Büeler, 2010). Specifically, in mammalian cells, cytosolic Parkin is selectively recruited to dysfunctional, depolarized mitochondria upon dissipation of ΔΨm by chemical uncoupler CCCP (carbonyl cyanide *m*-chlorophenyl hydrazone), then promoting their autophagic-lysosomal-mediated degradation. The Parkin translocation to depolarized mitochondria requires the activity of PINK which – in basal conditions – constantly undergoes a ΔΨm-dependent import into these organelles followed by specific maturation mediated by intrinsic protease(s), including matrix metalloproteinase (MMP), presenilin-associated-rhomboid-like (PARL), matrix-oriented AAA protease (m-AAA), and caseinolytic peptidase XP (ClpXP). Conversely, in response to low potential, full-length PINK is not imported/cleaved but rapidly stabilized, accumulating thus on outer membrane TOM (translocase of the outer membrane) complex (Batlevi and La Spada, 2011; Ashrafi and Schwarz, 2013; Grenier et al., 2013) where it engages Parkin which, in turn, triggers the mitochondria deliver toward autophagic-lysosomal pathway. Pink indeed recruits, directly or indirectly, Parkin from cytosol in close proximity on depolarized mitochondria and critically promotes its E3 ubiquitin ligase activity, likely by Ser65-phosphorylation, in the initial step of mitophagy (Kondapalli et al., 2012; Shiba-Fukushima et al., 2012; Iguchi et al., 2013). Parkin recruitment to mitochondria induces ubiquitination of several targets such as mitochondrial fusion proteins mitofusins (Mfns1/2) and voltage-dependent-activated channel (VDAC; Gegg et al., 2010; Geisler et al., 2010) whose proteasomal degradation (Chan et al., 2011; Yoshii et al., 2011) provokes the fragmentation of the organelle followed by its engulfment by autophagosomes (Deas et al., 2011). In addition p97, an AAA+ATPase, also accumulates on mitochondria in a Parkin-dependent manner to promote the degradation of OMM proteins and then mitophagy (Tanaka et al., 2010). Concerning the role of Parkin-mediated ubiquitination of Mfns1/2 in promoting mitophagy, two different but not mutually exclusive models have been proposed. To start with, the degradation of Mfns1/2 by UPS might disperse the clustered mitochondria and facilitate their engulfment by autophagosomes (Chan et al., 2011). Secondly, the removal of this pro-fusion mitochondrial protein shifts the balance toward the fragmentation – which is crucial in triggering mitophagy (Twig et al., 2008) – likely by physically interfering with the formation of Mfns1/2 *trans*-homodimers needed for tethering of these organelles (Ziviani and Whitworth, 2010). Alternatively, Parkin-mediated degradation might remove several negative regulators localized on the mitochondrial surface, thereby unmasking a molecular signal for recruitment of depolarized mitochondria by autophagosomes (Chan et al., 2011). Interestingly, the Parkin-induced elimination of Mfns1/2 is necessary for mitophagy to occur as it prevents the activation of an inhibitory default pathway in which depolarized and fragmented mitochondria undergo a drastic conformational change to become large spheroids that are not recognized by autophagosomes (Ding et al., 2012).

A large number of studies performed in non-neuronal cells lines have demonstrated that PINK1/Parkin pathway affects the autophagy clearance of damaged mitochondria in several ways: (i) by shifting the balance between fusion and fission of the mitochondrial network, allowing fragmented mitochondria to be taken up in autophagic vesicles; (ii) by modulating mitochondrial motility in order to switch the movement of mitochondria toward the autophagosome- and lysosome-rich perinuclear area, where the probability of being taken up by autophagosomes [AVs (autophagic vacuoles)] is higher and (iii) by directly recruiting the autophagic machinery to damaged mitochondria (de Vries and Przedborski, 2013). Neuronal mitochondria also undergo continuous reshaping by paired fission/fusion, transport, biogenesis, and selective degradation (Vives-Bauza et al., 2010; Fischer et al., 2012; Sheng and Cai, 2012; Youle and van der Bliek, 2012), as in immortalized cells lines (Youle and Narendra, 2011). Experimental data have indeed proved that PINK1/Parkin pathway regulating the mitochondrial quality control is active in primary neurons, as in replicating cell types (Seibler et al., 2011; Van Humbeeck et al., 2011; Yu et al., 2011; Cai et al., 2012; Joselin et al., 2012; Koyano et al., 2013; McCoy et al., 2014). However, other studies carried out *in vitro* (Van Laar et al., 2011; Rakovic et al., 2013) as well as *in vivo* (Sterky et al., 2011; Yu and Lu, 2011; Lee et al., 2012; Vincow et al., 2013) have yielded controversial results concerning the actual existence of mitophagy in neurons, especially those regarding the recruitment of Parkin to dysfunctional mitochondria. Indeed, in HeLa and MEF (mouse embryonic fibroblast) cell lines a general depolarization induced by pharmacological treatment with the uncoupler CCCP or other mito-toxins – such as antimycin A, valinomycin, and rotenone known to be ETC inhibitors – results in a rapid, robust, and faithfully reproducible re-localization of Parkin to mitochondria, followed by their prolonged and selective autophagic clearance (Narendra and Youle, 2011; Youle and Narendra, 2011; Ding and Yin, 2012; Youle and van der Bliek, 2012; Ashrafi and Schwarz, 2013). Likewise, immortalized cybrid cell lines carrying lethal mtDNA deletion – which causes a dysfunctional ETC assembly with decreased membrane potential – have also been shown to exhibit mitochondrial recruitment of Parkin at the beginning of mitophagy (Gilkerson et al., 2012). Conversely, in cultured primary neurons, a frank depolarization-evoked translocation of Parkin to mitochondria appears to be more variable. Discrepancies on (i) different type, handling and culture conditions (caspase(s) inhibitor, glial layer, antioxidant B27) used; (ii) expression of endogenous physiological level of proteins or transfection of exogenous transgenes; (iii) cell type-specific bioenergetic profile; (iv) type of injury and duration of treatment are indeed all important features that should be taken into account in evaluating studies on mitophagy mechanisms undergoing in post-mitotic neurons. As further discussed, the mitophagy pathways has turned out to differ – both spatially and kinetically – in cultured primary neurons from those found in immortalized lines (**Table 1**) and, perhaps, it might also diverge in its physiological function of removal of dysfunctional mitochondria.

**Table 1:** Comparison of Pink–Parkin pathway between immortalized cells and post-mitotic neurons.

	Immortalized cells*	Primary neurons (iPS-derived neurons)	bibliography
Dependence on ATG family proteins	Yes	Yes	Cai etal. (2012)
Pink-dependence	Yes	Yes	Vives-Bauza etal. (2010), Seibler etal. (2011), Joselin etal. (2012), Rakovic etal. (2013), McCoy etal. (2014)
ΔΨ Loss	Yes	Yes	Joselin etal. (2012), Koyano etal. (2013)
Meantime of Parkin recruitment upon ΔΨ depolarization	30 min	4 h 12 h 6 h 24 h	Joselin etal. (2012), Van Laar etal. (2011), Seibler etal. (2011), Cai etal. (2012)
% cells with Parkin recruitment	90%	25% 30% 45% 70%	McCoy etal. (2014), Cai etal. (2012), +ZVAD, Koyano etal. (2013), Joselin etal. (2012), no B27
Ubiquitination of mitochondria prior mitophagy	Yes	Yes	Joselin etal. (2012), Koyano etal. (2013)
Smaller mitochondria size and perinuclear clustering	Yes	Yes	Yu etal. (2011), Cai etal. (2012)
Loss of mitochondria	Yes	No	Van Laar etal. (2011), Yu etal. (2011), Rakovic etal. (2013)
Endogenous Parkin recruitment	Yes	?	Cai etal. (2012), Rakovic etal. (2013)
Ambra1	Yes	?	Van Humbeeck etal. (2011), coIP Ambra1/Parkin
Hexokinase 2	Yes	Yes	McCoy etal. (2014)

*HeLa, HEK293T, SH-SY5Y, MEFs.

To this regard, the recruitment of endogenous Parkin to depolarized mitochondria has been first provided by Narendra et al. (2008), showing that an enhanced immunoreactivity level of Parkin is found by Western blotting on crude mitochondria-enriched fractions from rat cortical neurons treated for 1 h with CCCP, although its increase is not as much significant as in HEK293 (human embryonic kidney 293) cells exposed to similar experimental conditions. Afterward, another paper (Vives-Bauza et al., 2010) has proved that this event is strictly Pink-1 dependence since the CCCP-induced collapse of ΔΨm for 1 h is not longer associated with an endogenous relocalization of Parkin to mitochondria in primary neurons from PINK1 (-/-) mice, as well as in *PINK1* – siRNA silenced HeLa cell line. More recently another group (Wang et al., 2011) reports that, as in non-neuronal HEK293T cells, treatment up to 1 h with a complex III inhibitor such as antimycin A also induces in cultured rat hippocampal neurons the translocation of exogenously expressed YFP-Parkin on axonal mitochondria, significantly decreasing their size and shape. In contrast, no evidence of a frank translocation of overexpressed as well as endogenous Parkin has been found to occur, neither in distal axons and dendrites nor in cell bodies mitochondria, in primary cortical neurons when they are acutely depolarized with CCCP up to 6 h, regardless of a clear evidence of active mitophagic pathway in these *in vitro* cultures (Van Laar et al., 2011). In agreement with the dispensable role of Parkin in degrading damaged mitochondria *in vivo*, overexpressed Parkin-independent accumulation and/or elimination of these organelles are allowed to occur in dopaminergic neurons of MitoPark mice carrying a severe respiration dysfunction due to their DNA (mtDNA) loss in mitochondrial transcription factor A (TFAM; Sterky et al., 2011). However, only recently, it has been clearly shown that the neuronal mitophagic response to an acute, excessive mitochondrial stressor is a process not only temporally slow but also spatially regulated (Cai et al., 2012). Compared with non-neuronal cells, CCCP-induced translocation of Parkin rarely occurs in mature neurons within 6 h and Parkin ring-like structures surrounding fragmented mitochondria are only occasionally detectable as early as 12 h of treatment, becoming increasingly frequent only at 18 h. In addition, the gradual and delayed Parkin recruitment to neuronal mitochondria (i) is compartmentally restricted to the somatodendritic regions and (ii) is coupled to reduced anterograde transport of these damaged organelles ending in an efficient lysosomal degradation into soma after 24 h exposure to CCCP (Cai et al., 2012). Interestingly, the fact that CCCP exposure does not initiate Parkin translocation and/or mitophagy in primary neurons up to 6 h might be in part explained because this process is regulated, to some extent, by bioenergetics dynamics since mammalian cells with a metabolism essentially glycolytic – if forced into OxPhos respiration – also recapitulate these effects, in a similar way of terminally differentiated neurons (Kanki and Klionsky, 2008; Van Laar et al., 2011). Indeed when glucose-fed HeLa cells were compelled to rely on mitochondrial oxidative phosphorylation in galactose/glutamine media – a culturing condition mimicking neuronal metabolism because it favors utilization of the citric acid cycle and oxidative phosphorylation over lactic acid-generating glycolysis for ATP production – they failed to recruit Parkin to their mitochondria upon CCCP-depolarization (Van Laar et al., 2011). The finding that Parkin translocates to depolarized mitochondria in glycolytic HeLa cells, which are grown in glucose-based media, but not in HeLa cells forced to rely on mitochondrial respiration, being grown in glucose-free media supplemented with galactose/glutamine, clearly shows that the induction of mitophagy is prevented by augmenting the cellular energy dependence on mitochondrial metabolism (Van Laar et al., 2011). Further, neither a robust association of overexpressed Parkin with mitochondria nor co-localization of these organelles with autophagic LC3 marker are clearly detected in cortical primary neurons at 1 h even after co-treatment with an ETC inhibitor such oligomycin which, blocking the reverse hydrolysis activity of mitochondrial ATP synthase, enable these *in vitro* cultures to temporarily hinder the massive energetic ATP drop caused by the CCCP-induced depolarization. Then again, the ATP loss induced by CCCP-depolarization is not the sole determinant in the inhibited Parkin translocation to mitochondria – and consequently in the induction of mitophagy – in oxidative phosphorylation-dependent neurons when compared to glycolytic HeLa cell lines (Van Laar et al., 2011). In view of these findings, it has been proposed that (i) the Parkin response is markedly suppressed in primary neurons in contrast to HeLa cells and that (ii) the rapid loss of ATP after a wide mitochondrial insult may prevent in neurons the full-scale Parkin-associated mitophagy, which may be quite important in conditions of slowly accumulating damages such as in aging-related human neurodegeneration. However, it is worth mentioning that even after 24 h, a selective damage to a small pool of mitochondria may still allow mitophagy processes since Parkin recruitment is seen in roughly 26.7% of neurons (Cai et al., 2012). Surprisingly, a restricted population of these mitochondria shows evidence of partial recovery of membrane potential – even after a prolonged depolarization induced by 24 h CCCP exposure – suggesting that a few, delayed bioenergetic compensatory changes occurring in primary neurons may yet permit these organelles to undergo elimination via autophagy (Cai et al., 2012). Finally a rapid (4 h) Parkin relocalization to mitochondria has been established to occur in mouse cortical neurons by exposing these cultures to a variety of mitochondrial damaging agents [i.e., CCCP, MPP^+^ (1-methyl-4-phenylpyridinium), and rotenone] in the absence of antioxidants, which are normally present in supplement B27 medium known to be routinely added to culturing medium of primary neuronal cultures. To this regard and consistently with a critical role of ROS in regulation of mitochondrial quality control, Joselin et al. (2012) report that mitochondrial translocation of Parkin is a ROS-dependent process in neurons because treatments with several ROS blockers can prevent at 6 h the recruitment of its overexpressed form in these cultures (as well as in MEFs). In addition, the same authors show that the loss of DJ-1 – a gene which is assumed to be critically linked to ROS management – can also trigger an accelerated stress-induced recruitment of Parkin to mitochondria facilitating thus their autophagic elimination via mitophagy. These findings not only undoubtedly confirm the active presence of Parkin/PINK1-mediated pathway in quality control of neuronal mitochondria but also (i) indicate that neuronal culturing conditions containing antioxidant supplements can prevent the pharmacologic effect of mitochondrial uncouplers and (ii) provide a possible explanation of the why several groups (Van Laar et al., 2011) are not able to detect significant Parkin translocation in neurons upon CCCP-depolarization. Interestingly TIGAR (TP53-induced glycolysis and apoptosis regulator), a biphosphatase which lowers the intracellular level of fructose-2,6-biphosphate (Fru-2,6-P2) with consequent inhibition of glycolysis and an overall decrease of intracellular ROS via increased production of NADPH through the pentose phosphate shunt, has also been recently identified as a novel negative regulator of neuronal mitophagy. In fact, antisense-mediated inactivation of TIGAR induces a complete normalization of impaired mitochondrial metabolism (morphology, complex I and III activity) in pink^-^^/^^-^ zebrafish with ensuing rescue of dopaminergic neurons, suggesting that the manipulation of its physiological level can be a promising novel target for disease-modifying therapy in PINK1-related PD (Flinn et al., 2013). Finally, in favor with the proposal that the Pink1/Parkin is dependent on bioenergetic status (Van Laar et al., 2011), an elegant paper has recently reported that the apparent discrepancy in depolarization-induced relocalization of Parkin to mitochondria of primary neurons could be ascribed to their different amount of hexokinase activity (HK), an enzyme which catalyzes the conversion of glucose to glucose-6-phosphate. In agreement with their previous experiments in HeLa cells, McCoy et al. (2014) find that overexpression of wild-type but not kinase-inactive HK2 promotes the increase in mitochondrial translocation of overxpressed Parkin upon 6 h CCCP-depolarization, even in the presence of B27 in culturing medium. Data from these authors show in fact that (i) the activity of glycolytic hexokinase is upstream of Pink-1 and is crucial for recruitment of overexpressed Parkin to mitochondria in cortical primary neurons upon 6 h treatment with CCCP, acting thus as a modifier of Parkin relocalization to these organelles; (ii) the Parkin relocalization to mitochondria in these cultures in response to depolarizing conditions requires both ATP synthesis and active Pink1 signaling (McCoy et al., 2014).

An important point that needs also to be taken into account is that the type of insult may also impose the outcome of neuronal response to mitophagy. The mitochondrial complex III inhibitor, antimycin A, has been recently reported to trigger, up to 1 h, a rapid relocalization of Parkin to axonal mitochondria in primary rat hippocampal neurons (Wang et al., 2011). Conversely, in the same experimental conditions of overexpression and presence of B27, a retarded and sometimes insignificant Parkin-associated mitophagy has been described to occur at 12–24 h upon CCCP-depolarization throughout the whole neuronal body, including the soma and axonal compartments (Van Laar et al., 2011), or only in cell bodies and in dendrites but not in axons (Cai et al., 2012). Contradictory results in dopaminergic neurons derived from induced pluripotent stem cell (iPS) cells have also showed that, although recruitment of both endogenous and overexpressed Parkin to mitochondria is detectable in these *in vitro* cultures after 12 h exposure to another agent depolarizing such as valinomycin, this translocation is accompanied by no significant degradation of mitochondrial proteins located at the outer or inner membranes and at the matrix in contrast to a net reduction in mitochondrial DNA copy numbers (Seibler et al., 2011; Rakovic et al., 2013). To start with, it is worth noticing that a prolonged incubation with a chemical uncoupler in absence of caspase inhibitors – whose usage is conversely advised by Cai et al. (2012) – may promote apoptosis in these cultures. Secondly, the bioenergetics of cultured differentiated iPS cells requires still to be fully analyzed as it might be more different from that of primary neurons. Interestingly, in addition to a diminished or delayed translocation of Parkin to mitochondria in response to depolarizing CCCP, neurons show also a different temporal threshold for commitment of mitophagy in comparison to immortalized cell lines. In fact, it has very recently been demonstrated that redistribution of the cardiolipin from inner mitochondrial membrane (IMM) to OMM of damaged mitochondria can act as signal in cargo selection for their autophagic elimination and that injured neurons exhibit a larger percentage in membrane externalization of this phospholipid when compared to CCCP-treated HeLa cells (Chu et al., 2013).

However, it’s noteworthy that Parkin can regulate the mitochondrial physiology in neurons along different ways which are beyond their selective elimination via autophagy and do not necessarily require its stable recruitment to them such as (i) promoting their biogenesis (Shin et al., 2011) and optic atrophy 1 (OPA-1)-dependent integrity (Müller-Rischart et al., 2013) via activation of nuclear transcription factors; (ii) controlling their axonal trafficking (Wang et al., 2011; Liu et al., 2012b); (iii) suppressing apoptosis via Bax ubiquitination (Johnson et al., 2012); (iii) regulating the selective turnover of respiratory chain proteins (Vincow et al., 2013), likely by chaperone-mediated extraction followed by proteasomal degradation (Margineantu et al., 2007) and/or by direct transport of selected cargo to lysosomes through mitochondria-derived vesicles (Neuspiel et al., 2008; Soubannier et al., 2012).

## THE PINK/PARKIN PATHWAY IN NEURONS: MORPHOLOGY AND AXONAL TRANSPORT OF MITOCHONDRIA, NUCLEATION OF NEW PHAGOPHORES THROUGH DIRECT AMBRA1 RECRUITMENT ON MITOCHONDRIA

As far as the role of PINK1/Parkin pathway on mitochondrial morphology in neurons, evidence have demonstrated that PINK1 knock-down leads to elongation of mitochondria in rat hippocampal as well as in dopaminergic primary cultures while overexpression of either PINK1 or Parkin results in an increased number of mitochondria that are smaller in size (Yu et al., 2011). Quite the opposite, studies carried out in cell lines such as HeLa (Exner et al., 2007), SH-SY5Y (Lutz et al., 2009; Dagda et al., 2011), and N27 (Cui et al., 2010) have reported that mitochondria are found to be fragmented upon knock-down of PINK1 or Parkin and that this fragmentation is likely to be the result of an excessive fission because it is accompanied by an increase in mitochondrial fission proteins paralleled by an inverse decrease in their fusion proteins (Cui et al., 2010). However, no useful information are further provided by data from PD patient-derived fibroblasts carrying the PINK1^Q126P^ or PINK1^G309D^ pathogenic mutations which show no evident and significant changes in mitochondria morphology, although there is a slight tendency toward fragmentation in comparison with related control subjects (Exner et al., 2007). Nevertheless, a recent paper (Cronin-Furman et al., 2013) reports that the expression of three PD patients-derived mitochondrial DNAs in independent lines of cytoplasmic hybrid (cybrid) neural cells can provoke differential effects on mitochondrial morphology (from rod-like to swollen, fragmented, and globular shape), on movement of these organelles along axonal processes and on their oxygen consumption, assessed as basal measurements of oxygen consumption rate (OCR). Furthermore, neurons are able to activate pathways that affect mitochondrial mitophagy differently from those operating in replicating, non-neuronal cultures, even if in response to an identical insult. Indeed, when primary cortical neurons are exposed for 12 h to camptothecin in order to mimic the chronic genotoxic oxidative stress occurring in neurodegenerative diseases (Lin and Beal, 2006), a significant mitochondrial elongation with a suppressed expression of fission protein Drp1 and Parkin is clearly detectable in these in vitro cultures, in contrast to the opposite effect of other forms of stress (i.e., staurosporine and glutamate treatment) which are able to induce a marked fragmentation of these organelles. Interestingly, camptothecin-induced DNA damaging in non-neuronal cell lines – such as fibroblasts – does not evoke any significant mitochondrial elongation, providing the possible existence of intrinsic cell-specific differences in biological response of these organelles to exposure to similar stressor (Wang et al., 2013). Noteworthy, more and more compelling evidence outlines an interesting role of Parkin in managing the mitochondrial dynamics in response to insults of different severity. On one hand, Parkin can exert adaptive effects upon damage of neuronal mitochondria since, when these organelles are irreversibly injured in response to severe stress, can promote their mitophagic elimination mainly via K63-linked ubiquitination. On the other hand, under a moderate insult conditions causing only minor defects to these organelles, Parkin can support the maintenance of their integrity by activating specific nuclear signaling pathway, for instance the stimulation of their biogenesis (Shin et al., 2011) via NF-κB-dependent transcription of reshaping OPA-1 protein (Müller-Rischart et al., 2013). In the same way the long-term, neuron-specific overexpression of Parkin can positively modulate the mitochondrial dynamics/activity, likely by promoting their biogenesis via the transcriptional co-activator PGC-1α, extending thus the lifespan of the whole organism during the physiological aging (Rana et al., 2013).

Another recently identified aspect of mitochondrial biology regulated by the PINK1–Parkin pathway in neurons is the axonal transport of these organelles (Wang et al., 2011; Liu et al., 2012b). That a proper control in the functional connections between mitochondrial fission–fusion dynamics and axonal transport is critical for sustaining the neuronal survival has been largely documented (Frederick and Shaw, 2007; Saxton and Hollenbeck, 2012), as proved by the fact that inhibition of mitochondrial fission protein Drp1 greatly reduces the number of these organelles in synaptic terminals (Verstreken et al., 2005; Kageyama et al., 2012). In fact, when Drp1 is deleted in post-mitotic neurons such as in cerebellar PCs, mitochondria appear elongated like swollen spheroids which accumulate oxidative damage, become respiration-incompetent and co-localize with mitophagic markers (i.e., LC3 and p62/sequestosome 1, p62/SQSTM1) and ubiquitin. Interestingly the activity of Parkin appears dispensable for mitochondrial ubiquitination in Drp1KO PCs, suggesting that other E3 ubiquitin ligases may also operate in the quality control of neuronal mitochondria (Kageyama et al., 2012). Likewise, the deficiency of Mfn2 in PCs also causes an anomalous development/maturation of the dendritic arborization which exhibits few spines generally lacking mitochondria which are, in contrast, mainly distributed in cell body (Chen et al., 2007). Furthermore, a large fraction of the mitochondrial mass resides in distal axons and dendritic processes – far away from the soma where lysosomes are localized (Holtzman and Novikoff, 1965) – suggesting thus that mitochondrial degradation in neurons is likely to require a long-distance retrograde transport of these organelles toward the cell body (Ashrafi and Schwarz, 2013). However, even if still now it is not completely clear when malfunctioning mitochondria are actually retrieved from axon, an engulfment of these depolarized organelles has been also described to occur locally in neurons within autophagosome before their transport to soma (Wang et al., 2011). Additional supports in favor of a causal link between mis-regulation of mitochondrial dynamics and neurodegeneration have been recently put forward by the finding that the coordinated ability of the Pink1/Parkin pathway in immobilizing unhealthy and faulty mitochondria prior their autophagic elimination has been evolutionary conserved (Wang et al., 2011). To this regard, in *Drosophila* motor neurons as well in primary rodent hippocampal neurons, the overexpression of PINK1 or Parkin causes a general decline of axonal motility of mitochondria in association with the loss of Miro, a mitochondrial GTPase interacting with the adapter protein Milton which, in turn, binds the kinesin motor protein KIF5 (Hirokawa et al., 2010). Indeed, upon PINK1 or Parkin overexpression or Parkin-induced recruitment to mitochondria, Miro is ubiquitinated by Parkin and targeted for proteasomal degradation so forcing the disassembly of the Miro/Milton/kinesin complex which, in turn, quarantine the movement of damaged mitochondria prior to their selective clearance. The ability of PINK1/Parkin pathway in preventing mitochondrial movement prior to their clearance is an important step in assuring a proper quality control of these organelles in neurons. In fact sequestration and/or eventual engulfment of dysfunctional mitochondria would prevent their harmful accumulation at high-energy demand regions of the neurites thus (i) preventing further neuronal stress due to local ROS production; (ii) enabling the intracellular pool of these organelles to be replenished by healthier ones (Wang et al., 2011; Liu et al., 2012b). Interestingly, the existence of an active Pink1–Parkin pathway in primary neurons may reflect the exceptional challenges for neuronal populations in supplying their distal processes with healthy mitochondria which locally provide sufficient energy and Ca^+^-buffering capacity needed for the synaptic transmission. At the same time, an active Pink1–Parkin pathway could also explain why mutations in their genes are critically involved in the etiology of PD affecting dopaminergic neurons of substantia nigra, which show high rates of calcium influx and a marked susceptibility of dopamine oxidation (Surmeier et al., 2010). Alternatively, it is possible that the PINK1/Parkin pathway may also regulate the mitochondrial transport through potential interaction of Parkin with microtubules (Yang et al., 2005) or that the lysosomal degradation could be completed outside the soma, since mitochondrial markers have also been detected occasionally in axonal autophagosomes (Maday et al., 2012). Remarkably, it is particularly striking that Mfn2, a disease gene involved in CMT neurodegeneration that is well known for having a key role in Parkin-dependent mitophagy (Ziviani et al., 2010 ), facilitates the axonal transport of mitochondria in both directions by forming a complex with Miro (Baloh et al., 2007; Misko et al., 2010).

Finally, it has recently reported that the direct interaction between endogenous Parkin and Ambra1, a positive regulator of the Beclin-1-dependent autophagy (Fimia et al., 2007), enhances the dynamics and efficiency of Parkin-dependent clearance of neuronal mitochondria (Van Humbeeck et al., 2011), by stimulating the peri-mitochondrial nucleation of new phagophores and their consequent engulfment by autophagosomes. In details, endogenous **Parkin** and Ambra1 coimmunoprecipitates from HEK293 cells, SH-SY5Y cells, and adult mouse brain and their interaction is strongly increased during prolonged mitochondrial depolarization; although there’s no clear evidence for ubiquitination of Ambra1 by **Parkin**. Ambra1 is not required for **Parkin** translocation to depolarized mitochondria but it critically contributes to Parkin-mediated mitophagy, by locally stimulating the activity of the class III PI3K complex (Beclin-1, Vps34, p150, and the autophagy-specific subunit Atg14) that is essential for the formation of new phagophores (Tooze and Yoshimori, 2010). Thus, Ambra1 recruitment to mito-aggresomes contributes to the spatially restricted, selective nature of mitophagy, allowing the controlled engulfment of dysfunctional mitochondria but sparing the rest of the cell from unwanted autophagic degradation (Van Humbeeck et al., 2011). Interestingly, under normal conditions, a pool of Ambra1 is docked by Bcl-2 at the mitochondria, inhibiting its autophagic function. After induction of the autophagy, mitochondrial Ambra1 is released from Bcl-2, enabling it to bind to Beclin-1 in order to initiate the phagophore formation (nucleation) at the mitochondrion (Strappazzon et al., 2011). However, whether Parkin and Ambra1 are degraded along with the mitochondria or dissociate from mitochondria after setting the mitophagic process, as well the detailed biochemical basis for their increased binding upon mitochondrial depolarization, are all important questions that remain to be still addressed.

## DIFFERENT REGULATION OF MITOCHONDRIAL DYNAMICS WITHIN SOMA AND NEURITIC PROCESSES IN NEURONS

**FIGURE 4:**
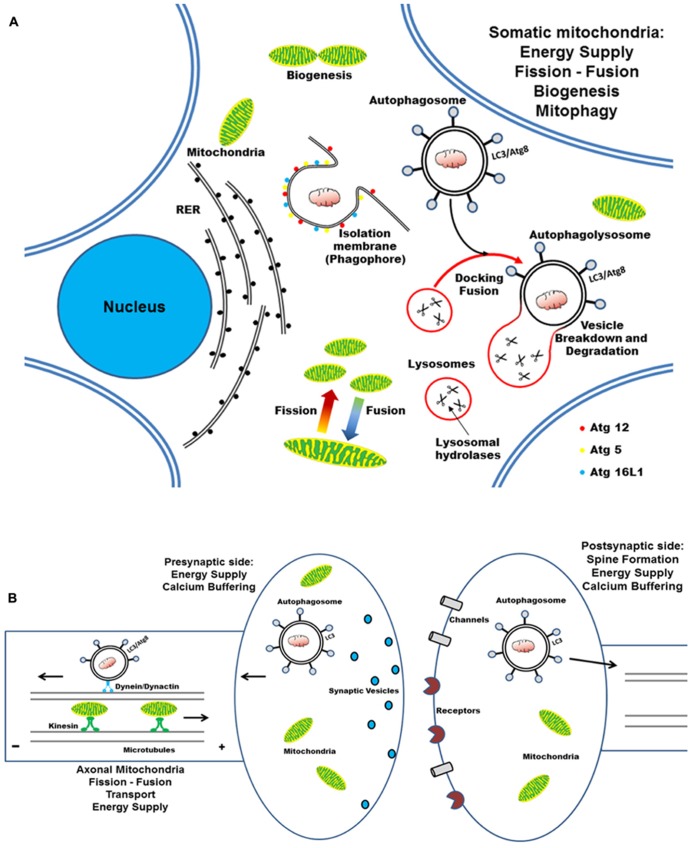
**Cartoon illustrating the mitochondrial turnover which copes with the compartmentalized and distinct energetic requirements in the cell body, axon, and synaptic compartments of post-mitotic neurons. (A)** In neurons, mitochondria travel long distances from the cell body out to distal dendritic and axonal terminals, where they subserve the ATP production and calcium homeostasis. The dynamic processes of biogenesis, fusion–fission regulate the mitochondrial function and quality control, by allowing them to adapt to spatial–temporal changes in cellular energy requirements. Selective autophagy begins with the nucleation of an isolation membrane (phagophore) which surrounds the damaged mitochondria to be degraded. RER could serve as membrane donors for autophagosome formation and the elongation of nascent double-membraned autophagic vesicle requires the coordinated assembly of Atg12–Atg5–Atg16L complex and LC3–PE conju-gates. **(B)** Newly formed autophagosomes move along microtubules in two directions – as a result of the opposing activities of the minus-end-directed motor protein dynein/dynactin and a plus-end-directed motor kinesin – and, finally, concentrate in perinuclear region (close to centrosome) where fuse with the lysosomes. Degradation of autophagosomal mitochondrial cargoes is then achieved by the acid hydrolases and the cathepsin proteases that are present in the lysosomal lumen.

A functional specialization into different sub-cellular compartments occurs in post-mitotic neurons since axon and dendrites, and not only the soma, exhibit unique biological and bioenergetic needs such as localized synthesis and degradation of proteins (Steward and Schuman, 2003; Piper and Holt, 2004). Furthermore axon and dendrites have each one their own specific transport mechanisms (Hirokawa et al., 2010; Namba et al., 2011), calcium regulation as well ER functions and localized ATP requirement (Hollenbeck and Saxton, 2005; Mironov, 2009; Wang and Schwarz, 2009; MacAskill and Kittler, 2010). Consequently, the regulation of mitochondrial turnover is more complex in neurons than in other mammalian non-neuronal cells because it has to provide for different sub-cellular compartments and locally regulate changeable concentrations of calcium and ADP (MacAskill et al., 2009; Mironov, 2009; Hirokawa et al., 2010; Cai et al., 2011; **Figure 4**). Synaptic activity (Rintoul et al., 2003; Sung et al., 2008), the levels of nitric oxide (NO; Zanelli et al., 2006) and calcium homeostasis, likely via the EF domains of Miro which operates as calcium sensor (MacAskill et al., 2009; Wang and Schwarz, 2009), tightly facilitate the trafficking and recruitment of neuronal mitochondria to high energy-demanding sub-compartments, such as dendrites and terminal fields. In response to the variable energy requirement of dendrites and axons, neuronal mitochondria can be locally anchored by interacting with neurofilament and neuron-specific intermediate filaments (Toh et al., 1980; Hirokawa and Takemura, 2005; Winter et al., 2008) or with microtubules via syntaphilin anchor (Kang et al., 2008). Interestingly, mitochondrial dynamics in axon and in distal dendrites of healthy post-mitotic neurons are less frequent of that observed in other non-neuronal cell or in cell bodies (Jendrach et al., 2005; Berman et al., 2009). This conclusion is supported by the fact that live-imaging quantifications show unexpectedly that in primary cultured neurons the process of fusion occurs in only 16% of mitochondria per hour in contrast to COS7 and INS1 cells where a fission event frequently occurs within 100 s after a coupled fusion (Twig et al., 2008). Furthermore, in highly polarized neurons, the mitochondrial fission/fusion balance is singularly regulated in different sub-cellular compartments depending on local energetic requirements in order to produce mainly elongated organelles in the somatodendritic compartment and more fragmented ones in distal axons (Overly et al., 1996; Popov et al., 2005). For instance synaptic mitochondria, which are localized at docking sites, significantly differ from other non-synaptic mitochondria as they are long-lived, undergo an increased oxidation during aging (Vos et al., 2010; Du et al., 2012) and contain higher levels of the matrix protein cyclophilin D which makes them more susceptible to calcium insult (Brown et al., 2006; Naga et al., 2007). These studies could also explain why the selective vulnerability of synapses (“dying-back” degeneration) is an early prominent characteristic of many human neurodegenerative disorders in which damage may begin at neuron terminals in the absence of any change in the cell body (Wishart et al., 2006; Bettini et al., 2007). Importantly, synaptic mitochondria are largely presynaptic (Gray and Whittaker, 1962; Nicholls, 1993) and residing mitochondria locally support the ATP synthesis and Ca^2^^+^ buffering in addition to the neurotransmitter synthesis and catabolism. Considering that (i) numerous neurodegenerative diseases are characterized by iron accumulation (Oshiro et al., 2011) which plays a crucial role in NMDA/NO-mediated neurotoxicity (Cheah et al., 2006; Chen et al., 2013); (ii) iron chelation is a strong Pink/parkin-independent activator of mitophagy (Allen et al., 2013), it has been proposed that an umbalance in homeostasis of this ion might affect in neurons the mitochondrial quality control processes (Allen et al., 2013). Interestingly, using primary cortical neurons which are chronically exposed to non-lethal low-concentration of rotenone, it has been proved that mitochondrial dynamics can be differentially affected in neurons over time and in specific sub-cellular regions. In fact, evidence outlines that the rates of fission and fusion of these organelles can modify during the *in vitro* cultures maturation (from 7 to 24 days of age) with changes which precede any sign of cell death, providing thus the existence of an important temporal window for new therapeutic opportunities prior to the onset of irreversible neurodegenerative phenomena. Besides, compartments-specific compensatory changes (i.e., homeostatic biogenesis) can be also possible in neurons in a attempt to balance the accumulating mitochondrial toxicity as demonstrated by the finding that the density of these organelles increases in more vulnerable and early affected distal neurites, prior to significant changes in cell bodies (Arnold et al., 2011). Furthermore, the morphology and function of ER, which plays a key role in mitochondrial fission (Friedman et al., 2011), may differ in somatodendritic compartment compared to distal axons (Ramírez and Couve, 2011) and the specialized sub-cellular contacts between ER and mitochondria [i.e., MAM (mitochondria-associated ER membrane)] involved in lipid synthesis and Ca^2^^+^ handling are also localized in hippocampal neurons at synapses to sustain locally their integrative activities and integrity (Hedskog et al., 2013). Finally, mitochondrial biogenesis occurs in neurons mainly in the soma (Davis and Clayton, 1996; Saxton and Hollenbeck, 2012) and the activation of its regulatory nuclear transcription factors – such as peroxisome proliferator-activated receptor gamma coactivator 1α (PPARGC1A/PGC-1α) and NFR-1/2 is controlled by neuronal activity (Yang et al., 2006; Scarpulla, 2008).

## CONCLUSION

Neurons – as well other non-neuronal cellular types – appear to be endowed by a common, basic molecular machinery involved in selective elimination of damaged mitochondria via an autophagic-lysosomal pathway named mitophagy. However, due to their specific morphological and bioenergetic necessities, differentiated neuronal populations display peculiar differences in mitophagic processes compared to other replicating cells and, sometimes, among the different brain regions. Increasing number of human neurodegenerative disorders are causally linked to an impaired mitochondrial turnover and two genes associated with autosomal recessive forms of PD, PINK1 and Parkin, have been recently found out to control the mitophagy also in primary post-mitotic neurons. Thus, the understanding how these unique neuronal features impinge on mitophagy and coordinated mitochondrial biogenesis will help to develop new disease-modifying approaches in future therapeutic interventions of neurodegenerative diseases.

## Conflict of Interest Statement

The authors declare that the research was conducted in the absence of any commercial or financial relationships that could be construed as a potential conflict of interest.

## Abbreviations

AD: Alzheimer’s disease
Aβ: amyloid-β
ALS: amyotrophic lateral sclerosis
Ambra1: activating molecule in Beclin-1-regulated autophagy 1
AMPK: 5′-AMP-activated protein kinase
Atg: autophagy-related gene
BAX: Bcl-2-associated X protein
CCCP: carbonyl cyanide *m*-chlorophenyl hydrazone
ClpXP: caseinolytic peptidase XP
DJ-1/PARK7: Parkinson protein 7
Drp1: dynamin-1-like protein
E3: ubiquitin ligase
ETC: electron transport chain
FIP200: focal adhesion kinase (FAK) family interacting protein of 200 kDa
HD: Huntington’ disease
HDAC6: histone deacetylase
HK: hexokinase activity
HSP90–Cdc37: heat shock protein 90–cell division cycle 37
IDE: insulin-degrading enzyme
IMM: inner mitochondrial membrane
iPS: induced pluripotent stem cell
KIF5: kinesin family motor protein 5
LC3: microtubule-associated protein 1A/1B-light chain 3
LIR: LC3 interacting region
m-AAA: matrix-oriented AAA protease
Mfns1/2: mitofusins 1/2
Miro: mitochondrial Rho GTPase 1
MMP: matrix metalloproteinase
mTOR: mammalian target of rapamycin
mTORC1: mTOR complex 1
mTORC2: mTOR complex 2
NFR-1: nuclear respiratory factor 1
Nix: NIP3-like protein X
OCR: oxygen consumption rate
OMM: outer mitochondrial membrane
OPA-1: optic atrophy 1
OxPhos: oxidative phosphorylation
p97: AAA-ATPase (also called VCP for valosin-containing protein)
PARL: presenilin-associated-rhomboid-like
PCs: Purkinje cells
PD: Parkinson’s disease
PE: phosphatidylethanolamine
PGC-1α: peroxisome proliferator-activated receptor α coactivator
PI3K: phosphoinositide-3 kinase
PI3P: phosphatidylinositol 3-phosphate
PIKK: phosphatidylinositol kinase-related kinase
PINK-1: PTEN-induced putative kinase 1
Raptor: regulatory associated protein of mTOR
RER: rough endoplasmic reticulum; ROS reactive oxygen species
SMURF1: E3 ubiquitin-protein ligase
SQSTM1: sequestosome 1
TFAM: mitochondrial transcription factor A
TIGAR: TP53-induced glycolysis and apoptosis regulator
TOM: translocase of the outer membrane
Ub: ubiquitin
UBA: ubiquitin binding domain
ULK-1: UNC-51-like kinase 1
UPS: ubiquitin–proteasome system
VCP: valosin containing protein
VDAC: voltage-dependent-activated channel
Vps: vacuolar protein sorting.
